# Positive Signature-Tagged Mutagenesis in *Pseudomonas aeruginosa*: Tracking Patho-Adaptive Mutations Promoting Airways Chronic Infection

**DOI:** 10.1371/journal.ppat.1001270

**Published:** 2011-02-03

**Authors:** Irene Bianconi, Andrea Milani, Cristina Cigana, Moira Paroni, Roger C. Levesque, Giovanni Bertoni, Alessandra Bragonzi

**Affiliations:** 1 San Raffaele Scientific Institute, Division of Immunology, Transplantation and Infectious Diseases, Milano, Italy; 2 Università degli Studi di Milano, Department of Biomolecular Sciences and Biotechnologies, Milano, Italy; 3 Institut de biologie intégrative et des systèmes (IBIS), Faculté de médecine et Pavillon Charles-Eugène Marchand, Université Laval, Québec, Canada; Medical College of Wisconsin, United States of America

## Abstract

The opportunistic pathogen *Pseudomonas aeruginosa* can establish life-long chronic infections in the airways of cystic fibrosis (CF) patients. Persistent lifestyle is established with *P. aeruginosa* patho-adaptive variants, which are clonal with the initially-acquired strains. Several reports indicated that *P. aeruginosa* adapts by loss-of-function mutations which enhance fitness in CF airways and sustain its clonal expansion during chronic infection. To validate this model of *P. aeruginosa* adaptation to CF airways and to identify novel genes involved in this microevolution, we designed a novel approach of positive-selection screening by PCR-based signature-tagged mutagenesis (Pos-STM) in a murine model of chronic airways infection. A systematic positive-selection scheme using sequential rounds of *in vivo* screenings for bacterial maintenance, as opposed to elimination, generated a list of genes whose inactivation increased the colonization and persistence in chronic airways infection. The phenotypes associated to these Pos-STM mutations reflect alterations in diverse aspects of *P. aeruginosa* biology which include lack of swimming and twitching motility, lack of production of the virulence factors such as pyocyanin, biofilm formation, and metabolic functions. In addition, Pos-STM mutants showed altered invasion and stimulation of immune response when tested in human respiratory epithelial cells, indicating that *P. aeruginosa* is prone to revise the interaction with its host during persistent lifestyle. Finally, sequence analysis of Pos-STM genes in longitudinally *P. aeruginosa* isolates from CF patients identified signs of patho-adaptive mutations within the genome. This novel Pos-STM approach identified bacterial functions that can have important clinical implications for the persistent lifestyle and disease progression of the airway chronic infection.

## Author Summary

*Pseudomonas aeruginosa* chronic infections cause persistent respiratory symptoms and decline of the lung functions in patients with cystic fibrosis (CF). Despite the continuous immune response of the host defense and the aggressive antibiotics treatment, bacterial persistence is anyhow established after an acute infection stage. *P. aeruginosa* establishes a permanent and detrimental relationship with the host by pathogenic variants different from the initially acquired strain. Currently, much is known about the bacterial factors needed for acute infections while the mechanisms involved in the colonization and persistence in chronic airways infection remain mostly unknown. The purpose of this study was to design a novel approach of genomics-based method for *in vivo* high-throughput screening to directly identify bacterial functions whose inactivation promotes airways long-term chronic infection. These studies may be relevant to the design of future drugs acting against chronic infections.

## Introduction

Persistent bacterial infections involving the opportunistic pathogen *Pseudomonas aeruginosa* are responsible for much of the morbidity and mortality caused by cystic fibrosis (CF). After causing an initial acute disease state, which is restricted by an immune response, *P. aeruginosa* establishes persistent infection and colonizes the host by evading immune surveillance [1] [2]. It has been shown that long-term colonization of the CF host is maintained by *P. aeruginosa* patho-adaptive lineages, which are clonal with the initially acquired strain and carried phenotypic variants [3]. A number of genetic mechanisms are responsible for generating clonal variants in *P. aeruginosa*, including acquisition or loss of genomic islands, genome rearrangements and recombinations [4]. Furthermore, point mutations lead to a wide spectrum of morphotypes with very minor genetic alteration [3]. Common mutations are consistently acquired by most CF strains as those in regulators of alginate biosynthesis (*mucA* and *algU*) [5] and virulence genes including motility (*rpoN*) [6], in the quorum-sensing regulator (*lasR*) [7] [8], in the type-III secretion system [9], in the multidrug-efflux pump (*mexA*) and in mutator phenotypes (*mutS*) [10]. Most of these *P. aeruginosa* mutants have morphotypes which are easy-to-follow by established *in vitro* assays [11] while other patho-adaptive mutations have been discovered by whole-genome comparison [3]. The sequence analysis identified 68 mutations in the *P. aeruginosa* late isolate when compared with its clonal early strain [3]. Most were single-base pair changes and many were predicted to result in a change or loss of protein function. Interestingly, virulence factors required for the initiation of acute infections were selected against during chronic infection. This indicates reduced virulence of the late strains with regard to their ability to provoke acute infection [12] [13]. This evolutionary scenario is similar to that of the genomes of other pathogens from other species. Genetic loss-of-function mutations confer enhanced fitness of the pathogen in a host-associated environment in *Escherichia coli* [14], *Haemophilus influenzae* [15] or *Helicobacter pylori* [16].

Despite the importance of *P. aeruginosa* clones with adapted virulence in the progression of CF airway disease, most patho-adaptative mutations and their role in the persistent lifestyle remain hidden in the genome due to the lack of genetic and functional tools for large-scale screens. The widening gap between the rapid progress in genome sequencing and the comparatively slow progress in the functional characterization of sequenced genomes represents a major concern to face the problem of *P. aeruginosa* chronic infection in patients with CF [17]. Furthermore, several different approaches, including signature tagged mutagenesis (STM), have been used to identify many bacterial genes required for virulence but they are restricted to certain stages of infection. STM is a genomics-based method for *in vivo* high-throughput screening based on transposon mutants tagged with a unique oligonucleotide. So far, STM has been based on a negative selection approach and applied to animal models of short-term acute infection. This approach selected and identified mutants with attenuated virulence in a variety of different pathogens [18], [19], [20]. According to the negative selection approach, mutants, present in the inoculum but not in bacterial pools recovered from short-term infected animals, are likely to be attenuated and therefore altered within virulence genes. On the contrary, a comprehensive screening of the bacterial genome for genes that identify the stage of chronic infection and can be the targets of phato-adaptative mutations has not been carried out. Progress in this field requires the development of a novel approach for the screening of STM libraries and sophisticated chronic infection models suited to directly identify functions whose inactivation promotes airways long-term chronic infection. Although several mouse models have been developed to mimic the chronic infection, the absence of CF-like lung disease, including long-term infection supported by a significant number of bacteria, has been disappointing [21]. We have recently reported that murine lung pathology associated with chronic infection induced by *P. aeruginosa* clinical strains isolated after years of colonization from CF patients, differs significantly to that of PAO1 reference strain [1]. In particular, exposure of murine airways to *P. aeruginosa* clinical strains embedded in the agar beads caused a long-term and higher rate of chronic infection than the challenge with the PAO1 prototype strain. The presence of patho-adaptive mutations in *P. aeruginosa* clinical strains and not in PAO1 may explain the different pathology in this model of murine chronic infection. Therefore, we designed a novel STM screening based on positive selection by using *P. aeruginosa* PAO1 STM library as source of mutants [22] and the validated agar beads mouse model of *P. aeruginosa* chronic infection mentioned above [23] [1]. This approach identified *P. aeruginosa* genes in PAO1 strain whose inactivation enhanced the establishment and maintenance of airways chronic infection similarly to clinical strains isolated from patients with CF. The features of the genes identified unmask novel strategies adopted by *P. aeruginosa* in the persistent lifestyle.

## Results/Discussion

### STM positive selection screening for *P. aeruginosa* mutations promoting long-term chronic airways infection

**Figure 1:**
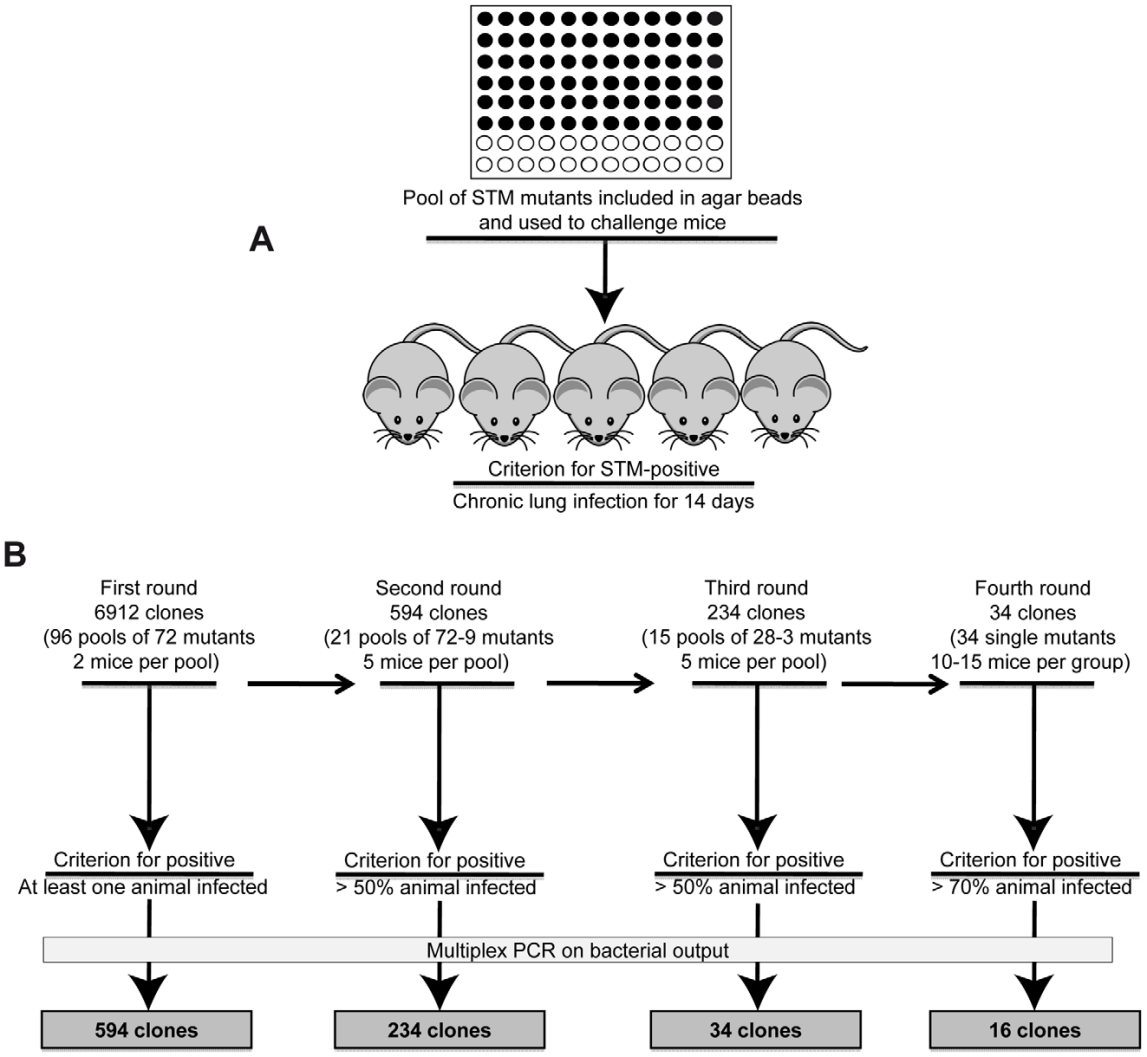
Implementation of the Pos-STM screening. **A**) First round of screening of a *P. aeruginosa* PAO1293 STM mini-*Tn5* library, consisting of 6912 mutants, arranged into 96 arrays of 72 mutants each [22]. The 72 *P. aeruginosa* mutants of each array were pooled, processed for agar beads preparation and injected into C57BL/6NCrlBR mice (input pool). After 14 days from challenge, mice were sacrificed, and bacteria recovered from lungs by plating homogenates. Bacterial cfu per lung and the percentage of infected mice were evaluated. **B**) In the first round of screening, clones that infected at least one of the two mice were identified as positive and selected for the following rounds of screening. For the second screening, we constructed 21 positive input pools with variable numbers of STM-mutants per pool, ranging from 72 to 9. Similarly, 15 positive input pools with variable numbers of STM-mutants per pool, ranging from 28 to 3 were constructed for the third screening. Mice were infected in second and third rounds of screening increasing to five the number of mice and selecting for more than 50% chronic infection. A total of 34 clones which passed the third round were tested singularly in the fourth round increasing the stringency for positive scoring and using 10–15 mice per group. STM mutants were identified by multiplex PCR. 16 mutants which increased significantly chronic infection (>70%) when compared to PAO1293 (12.5%) were considered positive Pos-STM mutants and analyzed further.

We wished to track patho-adaptive mutations promoting long-term *P. aeruginosa* infection in CF airways through the design of a STM screening based on positive selection (Pos-STM) *in vivo*. As tool to mimic the initial and progressive bronchopulmonary infection typical of CF patients, we used the validated agar beads-mouse model of chronic infection [23] [1]. The time course established previously with *P. aeruginosa* PAO1 [23] [1], and in this work with a PAO1 derivative PAO1293, demonstrated that both laboratory strains, given as doses ranging from 5×10^5^ to 5×10^6^ CFUs, induce low rates of acute mortality during the first three days (PAO1: 24.1% and PAO1293: 20%) and show capacity to establish chronic airways infection in some survivors (PAO1: 24.7% and PAO1293: 12.5%). Persistence *vs* clearance is taken as a simple read-out of an established chronic infection in the surviving mice at 14 days postinfection. To estimate bacterial levels in the chronic infection, surviving mice are sacrificed, lungs harvested, tissues homogenized and plated to evaluate bacterial load. The recovery of an output pool of more than 10^3^ CFUs of *P. aeruginosa* is an indication of chronic infection as described previously [23] [1]. Therefore, our criterion for the selection of mutations in *P. aeruginosa* promoting chronic infection was the screening for persistence rates higher than that of PAO1293 (12.5%) as described above. Indeed, we established, as criteria, to consider positive in screening assay mutant output pools of bacteria which persisted after several passages and at 14 days time points (**Fig. 1A**).

**Figure 2:**
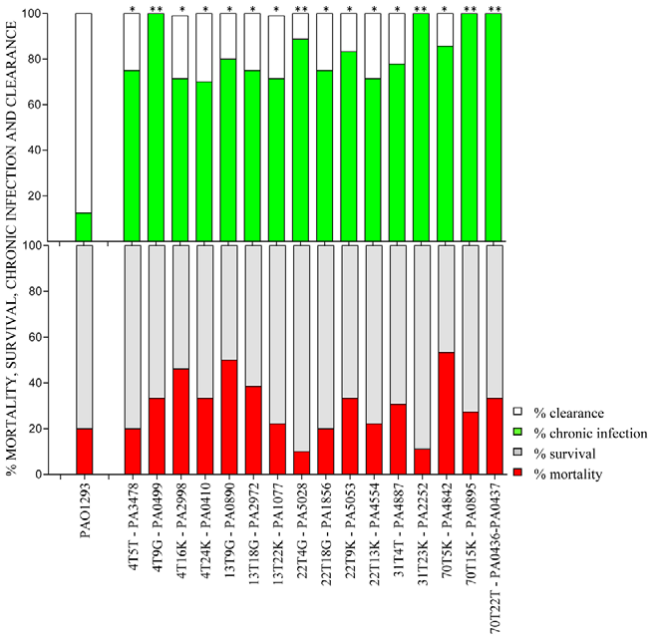
Virulence of *P. aeruginosa* Pos-STM mutants in a murine model of airways infection. C57Bl/6NCrlBR mice were infected with Pos-STM *P. aeruginosa* mutants embedded in agar beads. Mortality induced by bacteremia (red) and survival (grey) were evaluated on challenged mice. Clearance (white) and capacity to establish chronic airways infection (green) after 14 days from challenge were determined on surviving mice. The data show the percentage of mice infected with *P. aeruginosa* pooling together two to three independent experiments (PAO1293 *wt*: n = 10; STM mutants n = 10–15). Statistical significance by Chi square is indicated: *p<0.05, **p<0.01, ***p<0.001.

The source of mutants was a *P. aeruginosa* PAO1293 STM mini-*Tn5* library constructed previously [22], consisting of 6912 clones. In the first round of screening of the complete STM library (**Fig. 1B**), mice were challenged with 96 input pools of 72 mutants each. Since the large number of initial input pools, we decided to challenge only two mice per pool and to consider positive those challenges with at least one mouse with a chronic infection. Output pools of STM-mutants from positive challenges were further analyzed for clonal expansion of a given STM-mutant(s) by multiplex PCR (see Material and Methods for details) using as a template chromosomal DNA extracted from at least >10^3^ pooled bacterial colonies grown on agar plates. Following this first round of screening, we identified from the initial 96 input pools, 21 positive output pools in which we detected variable numbers of STM-mutants per pool, ranging from 72 (i.e. all mutants of the pool) to 9, respectively, giving a total of 594 mutants. The number of STM-mutants within a pool recovered after this initial screening must take into consideration the potential of cooperative behavior of virulence in mixed infection [1] [24]. The 594 STM-mutants from the first screening were assembled into 21 novel input pools and subjected to further rounds of *in vivo* screening. Since the input pools were smaller, we decided to increase the number of animals to five (per input pool) and selected for pools of STM mutants which persisted in at least >50% of surviving mice. In the second screening, we obtained 234 STM mutants, which distributed over 15 positive output pools with variable numbers of STM-mutants per pool, ranging from 28 to 3. To proceed further, the 234 STM-mutants resulting from the second screening were assembled from the original STM library into 15 novel input pools and subjected to further step of screening. As shown in **Fig. 1B**, the third round of screening decreased the number of STM mutants to 34. Finally, in the last round of screening, the 34 STM-mutants were tested singly in larger groups of mice ranging from 10 to 15 animals and with stringency for positive scoring of persistence increasing to >70% of surviving mice. Sixteen of the 34 STM mutants were confirmed as Pos-STM mutants, i.e. they caused a significant increase in percentage of chronic infection in surviving mice when compared to the PAO1293 *wt* (Chi square analysis: PAO1293 *wt* vs Pos-STM mutants 12.5% vs 70–100%, p<0.05) (**Fig. 2** **and Table S2** in **Text S1**). During the first three days of infection, STM-mutants caused low mortality with no significant differences compared to PAO1293 *wt* (Chi square analysis: PAO1293 *wt* vs Pos-STM mutants 20% vs 10–53.3%, p>0.2).

### Histopathological lesions of *P. aeruginosa* Pos-STM mutants promoting long-term chronic airways infection

**Figure 3:**
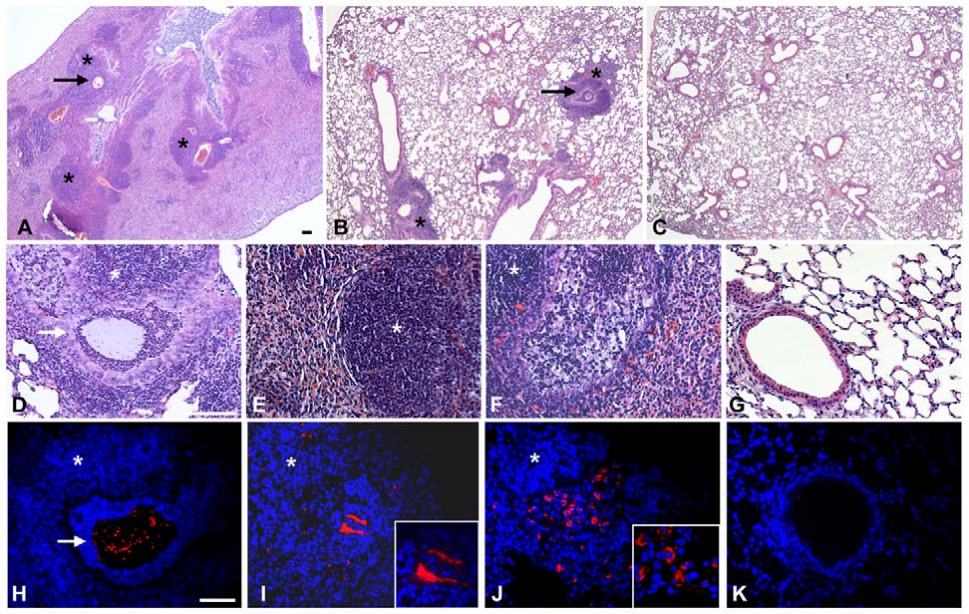
Murine lung histology and localization of *P. aeruginosa* Pos-STM mutants after 14 days. Mice were infected with 2×10^6^ cfu/lung of *P. aeruginosa* Pos-STM strains embedded in agar beads. In this panel, PA5053- *hslV* (**A and B**), PA2998-*nqrB* (**D and H**), PA4554-*pilY1* (**E and I**) and PA0499 (**F and J**) are taken as representative of the whole set of 16 Pos-STM mutants. Control mice were infected with PAO1293 *wt* (**C, G and K**). The lungs were stained with H&E (**A–G**) or with specific antibody against *P. aeruginosa* strains (red) (**H–K**). Counterstaining was performed with 4′,6-Diamidino-2-phenylindole dihydrochloride (DAPI) (blue) (**H–K**). Bronchi and pulmonary parenchyma are characterized by chronic lesion (*) infiltrated by macrophages, lymphocytes and neutrophils. Severity of lesions and lung involvement is heterogeneous in different lobes of the same mice (**A and B**). Bronchi contain massive granulocyte infiltration of bacteria and agar beads (arrow) (**D and H**) or bronchial wall is disrupted and the bronchial lumen is filled by foamy macrophages and bacteria presumably internalized by inflammatory cells (**F and J**). Bacterial macrocolonies are visible in the pulmonary parenchyma outside the beads (**E and I**). PAO1293 *wt* infected mice resolved the infection and inflammation (**C, G and K**). A–C: 2.5×; D–K: 20×; insert I–J: 63×. Confocal microscopy (**H–K**). Bars, 100 µm.

To assess clinical strain-traits of chronic infection, lung histopathology was performed on mice challenged singularly with the Pos-STM mutants for 14 days. The histopathological analysis of *P. aeruginosa* Pos-STM-induced chronic pneumonia indicates that in this model the lung was not totally compromised: the infection was pluri-focal and generally involved one or more lung lobes (**Fig. 3A**), whereas the others were unaffected or marginally involved (**Fig. 3B**). Bronchi were filled by agar beads surrounded by a massive neutrophilic infiltration (**Fig. 3D**) and the surrounding parenchyma was principally infiltrated by macrophages, lymphocytes and neutrophils (**Fig. 3E**). In other cases bronchial wall was disrupted with neutrophilic infiltration replaced by ‘foamy’ macrophages (**Fig. 3F**). The infected bronchi contained fewer and smaller agar beads at 14 days when compared to day 0, which were partially degraded by surrounding neutrophils and released bacterial cells. Immunofluorescence staining observed at confocal microscopy showed that the persisting bacterial cells were localized within the bronchial lumen inside the beads **(** **Fig. 3H** **)** and outside the beads as macrocolonies **(** **Fig 3I** **)**. Therefore, some of the persisting bacterial cells of the Pos-STM mutants were protected from the murine respiratory defense system by the biofilm formation and not by the beads. Furthermore, Pos-STM mutant cells were presumably found invading inflammatory cells **(** **Fig 3J** **)**. Severities of lesions in the lung were similar between all the Pos-STM mutants while most of the PAO1293 *wt* controls resolved the infection and inflammation (**Fig 3C, G and K**). Similar histopathological lesions were previously shown in murine lung infected for 14 days with late isolates from CF patients [1]. These results indicate that single gene inactivation in PAO1293 can favor the establishment of chronic infection reminiscent of the so-called “unique” strain lineages of *P. aeruginosa* pathogenic variants isolated from CF patients.

### Analysis of the Pos-STM mutants revealed transposon insertions within known virulence genes and other genes not previously associated to infection

Each Pos-STM mutant was subjected to mapping of the transposon insertion by sequencing of the mini-Tn5 flanking genomic regions (**Table S1** in **Text S1**). This first analysis individuated single insertions within a specific gene in 15 Pos-STM mutants (**Table 1**) and a single insertion into the intergenic region between *loci* PA0436 and PA0437 in the remaining Pos-STM mutant. However, a further test by Southern blotting to ensure that the Pos-STM mutants did not carry more than one transposon insertion showed that mutants 70T5K and 70T15K carried one additional mini-Tn5 insertion that eluded the previous analysis of mapping (**Table 1**).

**Table 1:** Genotypic and phenotypic characterization of Pos-STM mutants.

STM-mutant	Number of *Tn* insertions	PA number^a^	Gene name	Relevant phenotype^b^
4T24K	1	PA0410	*pilI*	Swimming and twitching defect
4T9G	1	PA0499	-	-
13T9G	1	PA0890	*aotM*	Increased biofilm formation
70T15K	2	PA0895	*aruC*	-
		nd^c^		
13T22K	1	PA1077	*flgB*	Swimming defect
				Decreased biofilm formation
22T18G	1	PA1856	-	-
31T23K	1	PA2252	-	-
13T18G	1	PA2972	-	Twitching defect
4T16K	1	PA2998	*nqrB*	-
4T5T	1	PA3478	*rhlB*	Increased biofilm formation
22T13K	1	PA4554	*pilY1*	Swimming and twitching defect
				Pyocyanin secretion defect
				Decreased biofilm formation
70T5K	2	PA4842	-	Pyocyanin secretion defect
		nd^c^		
31T4T	1	PA4887	-	-
22T4G	1	PA5028	-	-
22T9K	1	PA5053	*hslV*	-
70T22T	1	PA0436-PA0437 IR^d^	-	-

a *locus* ID according to Pseudomonas Genome Database_V2_ (www.pseudomonas.com).

b relevant phenotype of Pos-STM mutants compared to PAO1293 *wt*.

c not determined.

d intergenic region.

The 15 mini-Tn5 inserted genes encoded proteins from almost all functional classes (**Table S3** in **Text S1**): hypothetical, unknown, unclassified proteins (PA2972, PA4842, PA5028), motility and attachment (PA0410-*pilI*, PA0499, PA1077-*flgB*, PA4554-*pilY1*), putative enzymes (PA1856), transport of small molecules (PA0890-*aotM*, PA2252, PA4887), amino acid biosynthesis and metabolism (PA0895-*aruC*), energy metabolism (PA2998-*nqrB*), secreted factors (PA3478-*rhlB*), chaperones and heat shock proteins (PA5053-*hslV*). However, we noted that some insertions map into either the first or internal genes of certain operons, as listed in **Table S3** in **Text S1**, and polar effects on the expression of downstream genes could account for the observed phenotype. In the case of mutants 70T5K and 70T15K, the Pos-STM phenotype can be due to a combination effect caused by the double insertion and or recombination events. Finally, since the intergenic transposon insertion in the 70T22T STM mutant is between convergent PA *loci*, we speculate a negative effect of the insertion on transcription termination. Furthermore, inactivation of small non coding RNA gene(s) cannot be ruled out. Interestingly, previous report showed that eight different intergenic regions were mutated in the 96-month isolate from CF patients [3].

In a second step of analysis, the 16 Pos-STM mutants were tested for the following traits, known to play an important role in the *P. aeruginosa* pathogenesis of CF infections: motility, mucoidy, hypermutability, protease secretion, siderophore, hemolysis, autolysis, pyocyanin production, biofilm formation and LasR phenotype, consisting of visible accumulation of the iridescent intercellular signal 4-hydroxy-2-heptylquinoline due to loss-of-function inactivation of *lasR*. The results of these analyses are summarized in **Table 1** and **Table S4** in **Text S1**. No Pos-STM mutant showed mucoid and LasR phenotypes, or defects in protease secretion and siderophore production. On the contrary, a significant proportion of mutants in functions associated with motility emerged in this screening. In fact, three Pos-STM mutants carried insertions in PA0410-*pilI* and PA4554-*pilY1*, involved in type IV pilus biogenesis, and in PA1077-*flgB*, coding for the flagellar basal-body rod protein. Moreover, the Pos-STM mutant in PA2972 coding for an hypothetical protein was found to be defective in twitching motility. Our results, which indicated that a loss of motility favors the establishment and maintenance of chronic infection, are consistent with other studies. On the one hand, lack of motility is one of the phenotypes acquired by *P. aeruginosa* isolates over the course of CF airway infection and increases the risk of chronic infection in animal models [3] [1]. On the other hand, it was shown that the risk of bacteremia is increased by the presence of pili and flagella since they confer a selective advantage for spreading from the lung to other organs [25] [1] [26]. In addition to motility, flagella and pili also provide a ligand for phagocytic clearance [25]. Moreover, there is another striking consistency of our data with a previous *P. aeruginosa* STM screening in a polymorphonuclear neutrophil (PMN) phagocytosis assay [27]. In that case, two independent STM-mutants in *pilY1* arose from the screening since they showed higher survival rates (i.e. more resistant to killing by PMNs) than the wild type and any of the 3500 tested STM-mutants. In murine model, inactivation of *pilY1* impacted virulence promoting *P. aeruginosa* persistence in the airways [27]. Taken together, these consistencies validate our Pos-STM approach and strongly indicate its robustness for the identification of novel genes which impact the genetic adaptation of *P. aeruginosa* in CF airways.

Pyocyanin secretion defects were found in the PA4554-*pilY1* mutant, as expected from a previous report [27], and in the PA4842 mutant. Finally, regarding biofilm formation, PA4554-*pilY1* and PA1077-*flgB* mutants showed low levels of biofilm, respectively. This was not surprising given the involvement of flagella and pili in biofilm development by *P. aeruginosa* [28]. On the contrary, PA3478-*rhlB* and PA0890-*aotM* showed an improved ability to form biofilm. The remaining nine Pos-STM mutants showed no alteration in motility, pyocyanin secretion, and biofilm formation. As in the case of *pilY1* mutants, higher fitness to colonize and persist in airways may be multifactorial. This may also be true for PA3478-*rhlB* and PA0890-*aotM* mutants. One key factor for chronic infection could be the improved capacity to form biofilm. Other factors may be directly related to the inactivation of *rhlB* and *aotM* genes, respectively, as described below. The *rhlB* gene encodes RhlB rhamnosyltransferase [29], an enzyme involved in the synthesis of the surfactant mono-rhamnolipids (RLs) [30]. Among their roles, RLs have been shown to act as immune modulators and virulence factors. It has been demonstrated that purified RLs act directly on immune cells. For instance, they have been shown to induce direct neutrophil chemotactic activity, to stimulate the copious release of interleukin IL-8, granulocyte-macrophage colony stimulating factor and IL-6, and to induce the lysis of PMNs. Therefore, the inactivation of *rhlB* gene could attenuate the bacterium and allow it to evade the immune system. The reduced ability to induce IL-8 secretion was assessed for the PA3478-*rhlB* mutant (see below). Whereas, the improved fitness for chronic infection of PA0890-*aotM* mutant may be more closely-related to bacterial metabolism. In fact, the *aotM* gene codes for a component of the arginine/histidine ABC transporter, involved in the catabolism of arginine and controlled by ArgR and L-arginine under aerobic conditions [31]. Inactivation of *aotM* may stimulate the anaerobic catabolism of arginine by the arginine deiminase (ADI) pathway [32] and thus enhance the fitness for the colonization of and the persistence in the airways. The same effect may be possible for PA0895-*aruC* mutant. In fact, *aruC* codes for N-succinylglutamate 5-semialdehyde dehydrogenase involved in the aerobic arginine utilization via the arginine succinyltransferase (AST) pathway [33]. Remarkably, *P. aeruginosa* anaerobic catabolism of arginine can be relevant for colonization of CF lung which is typically an anaerobic niche [34]. Mutation-induced metabolic rearrangements could also involve other Pos-STM mutants. For instance, inactivation of PA1856, coding for a probable cytochrome c oxidase cbb3-type (subunit I), or PA2998-*nqrB*, coding for Na(+)-translocating NADH-quinone reductase subunit B, may rearrange the *P. aeruginosa* branched (micro)aerobic respiratory chain [35]. Furthermore, inactivation of PA2252, coding for a probable AGCS sodium/alanine/glycine symporter, or PA4887, coding for a probable major facilitator superfamily (MFS) transporter, may induce a metabolic reset better suited to colonize and persist in airways. Interestingly, another probable MFS transporter (PA2092) was mutated in the 96-month isolates from CF patients [3].

The significantly high number of Pos-STM mutants in genes related to metabolic functions strongly supports a key role of bacterial metabolic adaptation during chronic infections. This notion was raised previously [7] for mutations inactivating *lasR*, one of the most common targets of mutation in *P. aeruginosa* CF isolates [3]. The *lasR* loss-of-function mutations in these strains confer a growth advantage with particular carbon and nitrogen sources, including amino acids [7]. This growth phenotype was supposed to contribute to positive selection of *lasR* mutants within CF airways.

On the whole, as suggested previously by the analysis of *P. aeruginosa* isolates over the course of CF airways infection, the characterization of the panel of the Pos-STM mutants strongly indicates that chronic-adapted phenotypes reflect alterations in different aspects of the bacterium's biology. Remarkably, our Pos-STM screening did not detect mutants in functions associated with the alginate biosynthesis resulting in a mucoid phenotype, or with mismatch-repair systems giving rise to hypermutability. These two phenotypes usually appear relatively late in the chronic infection [10] [5] and it is possible they might not be critical for the establishment and the early stages of chronic infection. Furthermore, the Pos-STM screening may be affected by other factors limiting the technology as discussed in the conclusion below.

### Cellular invasion and stimulation of the host immune response by Pos-STM mutants

**Figure 4:**
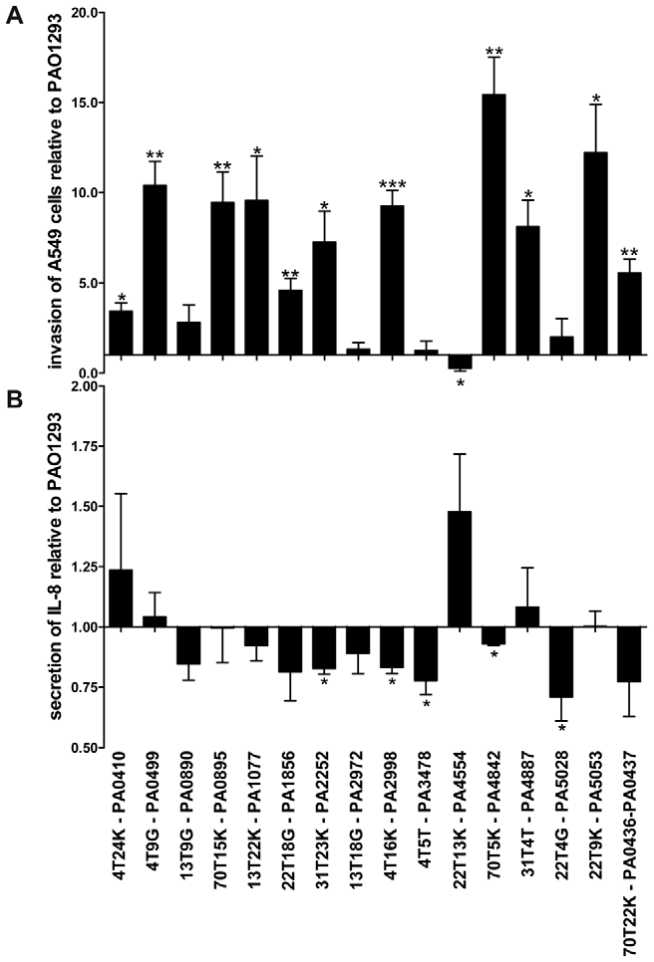
Stimulation of A549 with *P. aeruginosa* Pos-STM mutants. **A**) Fold of invasion relative to PAO1293 after 2 h of stimulation with Pos-STM mutants. **B**) IL-8 secretion was quantified by ELISA after stimulation with Pos-STM mutants for 2 h. Measurements were performed in triplicate. *p<0.05, **p<0.01, ***p<0.001 in the Student's t-test.

CF airway chronic infections occur with *P. aeruginosa* genetic variants and depend on the ability of bacteria to resist the host defense [1] [2]. For these reasons, in addition to the bacterial phenotypes analyzed above, we tested the ability of the Pos-STM mutants to invade human respiratory epithelial cells and to stimulate an immune response. As shown in **Figure 4A**, 11 of 16 Pos-STM mutants were found to be significantly more invasive than the reference PAO1293 *wt* strain. On the contrary, PA3478-*rhlB* and PA0890-*aotM* mutants were found to have a non-invasive phenotype. This feature may be related to the production of higher levels of biofilm by these strains. Other Pos-STM mutants showing limited invasiveness as PAO1293 *wt* were found in PA2972 and PA5028. Finally, PA4554-*pilY1* was found to be significantly less invasive in A549 cells than PAO1293 *wt*.

When the bacteria-induced IL-8 secretion was tested in A549 cells, most of the Pos-STM mutants were found to induce lower stimulation of IL-8 secretion. Significant differences were obtained for PA2252, PA2998-*nqrB*, PA3478-*rhlB*, PA4842, and PA5028 when compared with PAO1293 *wt* (**Figure 4B**). Therefore, the majority of mutants showed increased invasiveness and/or lower stimulation of IL-8 secretion. Together, these results indicate that invasion of host cells and/or lower stimulation of IL-8 secretion can be instrumental to the higher fitness to colonize and persist in CF airways.

Consistently with data obtained in *P. aeruginosa* clinical strains [2] and strategies used by a number of other bacterial pathogens [36], [37], [38], it is feasible that Pos-STM mutants acquired mechanisms for cellular invasion. Internalization in a host could benefit bacterial survival through evasion of host phagocytic- and antibody/complement-mediated killing mechanisms. Intracellular survival is a key component in the pathogenic cycle of a number of bacterial pathogens, including *Mycobacteria* spp., *Shigella flexneri*, *Salmonella* spp., *Legionella pneumophila* and *Burkholderia cenocepacia* [39] [40] [41]. Furthermore, in CF disease, decreased internalization of *P. aeruginosa* in CFTR deficient cells may increase bacterial lung burdens and contribute to establish biofilm-mediated chronic infection [42] [43].

Several lines of evidence indicate that, to permit long-term colonization, there has been selective pressure on pathogens to avoid triggering an intensive inflammatory reaction. Comparing lipid A and peptidoglycan of sequential strains isolated from CF patients, we recently showed that *P. aeruginosa* has evolved the capacity to evade immune system detection [2]. In this study, we extended this notion showing that the majority of Pos-STM mutants reduced the immune stimulatory potential. These findings emphasize further studies to establish whether *P. aeruginosa* adapted mutants evade immune system thus promoting survival and establishing favourable conditions for chronic persistence in CF patients.

### Pathoadaptive mutations in *P. aeruginosa* clinical strains from CF patients

Next, we asked whether the mutations observed in our Pos-STM approach are, to the some extent, representative of patho-adaptive mutations associated with *P. aeruginosa* infections in CF patients. To address this issue, we set out to sequence the 15 genes which were inserted in the Pos-STM mutants in a collection of longitudinal isolates from seven different CF patients; they include early strains isolated at the onset of chronic colonization and late strains collected over a period of up to 16.3 years [5]. Out of 15, we could obtain the full-length sequence of 14 genes. In fact, we were unable to amplify by PCR the full-length *pilY1* gene sequence throughout the collection of the *P. aeruginosa* longitudinally isolates. We attempted to amplify internal gene regions. The sequence analysis of the few PCR fragments that we obtained indicated several changes involving the *pilY1* gene. Thus, this strongly indicated that *pilY1* gene underwent extensive changes in the clinical isolates. Sequences analysis of the remaining genes by comparison between early and late strains revealed single base-pair synonymous and non-synonymous mutations in seven of 14 genes in six different clonal lineages (See **Table 2** and **Table S5** in **Text S1** for the complete panel of mutations). Non-synonymous mutations were found in five genes (PA0895-*aruC*, PA1856, PA2252, PA3478-*rhlB* and PA4887) from five different clonal lineages of CF patients (**Table 2**). A computational method for predicting the effect of each non-synonymous mutations on protein function [44] suggested that the non-tolerated changes in PA0895-*aruC*, PA3478-*rhlB* and PA4887 are likely to affect protein function (**Table S5** in **Text S1**). *P. aeruginosa* clonal isolates from a single time point after long-term chronic infection were heterogeneous, indicating a genetic diversification of a single strain during growth in CF airways as previously reported [3]. Overall, these data hint that some Pos-STM mutations, might recapitulate patho-adaptative mutations occurring in *P. aeruginosa* clinical strains. Previous studies have shown that well-known pathoadaptative mutations in *loci* including for instance *mucA* [5], *LasR* [7] or *mutS* [10] are common but not acquired by all *P. aeruginosa* isolates from CF patients. These observations have supported the notion that a number of genes in the genome are targets for patho-adaptative mutations promoting CF airways chronic infection, although most of these genes are mutated in only a fraction of bacterial strains isolated from infections. Therefore, the analysis of significant rates of the patho-adaptative mutations in Pos-STM genes identified in this study could require the study of broader collections of longitudinal *P. aeruginosa* isolates from CF patients.

**Table 2:** Gene sequence comparison between early and late *P. aeruginosa* isolates from CF patients.

Gene name	PA number	Gene function	Clinical strain*	No. of nonsynonymous mutations	aa mutation
*aruC*	PA0895	N-succinylglutamate 5-semialdehyde dehydrogenase	TR1 vs TR67	1	G197-V
*-*	PA1856	putative enzyme	AA2 vs AA43, AA44	2	V97 - A, I180 - M
			BT2 vs BT72, BT73	2	A97 - V, M180 - I
*-*	PA2252	probable AGCS sodium/alanine/glycine symporter	AA2 vs AA43	1	M4 - K
			KK1 vs KK72	2	R464 – H, N480 - T
*rhlB*	PA3478	rhamnosyltransferase chain B	KK1 vs KK71	1	H201 - Q
*-*	PA4887	probable major facilitator superfamily (MFS) transporter	SG1 vs SG57	2	K18 – V, K18 – V
			SG1 vs SG58	2	K18 – M, L212 - M

*Strains were collected at the onset of chronic colonization (numbered 1–2 per patient) or after 4–18 years of chronic airway colonization (numbered 43–73) [5] [1].

### Conclusions

For the first time in an animal model of chronic lung infection by *P. aeruginosa*, an STM approach based on positive selection is presented. The main objective of this work was to address the genetic adaptation within a defined genetic system of *P. aeruginosa* in CF airways through loss-of function mutations in virulence factors and regulators. As with typical STM, the Pos-STM is a screening assay and has limitations. For instance, Pos-STM can be biased by factors such as pooling of mutants giving intra-complementation between cells growing *in vivo*. Bacteria cells that are not necessarily enhanced in establishing chronic infection can be maintained by those that are. This can be overcome by using several rounds of screening and diminishing the number of mutants per pool as done here. Other limitations include the concentration of bacteria in the inoculum, the limited number of initial STM mutants (6912 mutants in this case). Obviously, the collection of STM mutants used is not representative of the complete genome but covers a small fraction of genes. Genes not in the STM collection will not be selected by Pos-STM. Other factors limiting this technology include the time points selected after infection to recuperate bacteria and the selective pressure for the bacterial growth in the agar beads mouse model. As a result, we identified 16 insertion mutations which lead to an increase in the fitness to promote chronic infection. This panel of mutants shows directly that *P. aeruginosa* establishes and maintains long-term chronic infection with patho-adapted variants. The reliability and robustness of our Pos-STM approach is supported by the high proportion of mutations in motility-related functions which are known key players in the pathogenesis of *P. aeruginosa*. Remarkably, our findings rigorously support the previous hypothesis that virulence factors essential for acute infection are lost when *P. aeruginosa* establishes long-term chronic infection. In addition to this, our screenings resulted in the identification of novel genes which were not previously associated to infection. Thus, our novel approach provides the basis to investigate the *P. aeruginosa* pathogenesis further and to extrapolate the results to other bacterial pathogens. The significantly high number of these genes linked with metabolic functions reinforces the notion of a key role played by *P. aeruginosa* metabolic adaptation during chronic infection. Furthermore, the behaviour of the majority of our Pos-STM mutants on host cells suggests that invasiveness and/or altered stimulation of the immune response influence the fitness to colonize and persist in CF airways. The fact that many of these bacterial functions have been missed in the other screenings, including large-scale DNA sequencing [3] and morphological phenotypes assays [3], emphasizes the gap of tools for profiling chronic infection and points to the need to perform this novel Pos-STM approach.

Finally, some of the genes of this STM screening were found to be mutated throughout a collection of longitudinal isolates from CF patients. This hints that they may be targets of patho-adaptive mutations during the *P. aeruginosa* microevolution during CF lung infection with major implication in the progression of the lung disease. The novel approach of positive-selection screening, described for the first time in this study, should find general applicability to other pathogens that enhance fitness in their host through patho-adaptive mutations and so provide a basis for more comprehensive understanding of chronic infection diseases.

## Materials and Methods

### Ethics statement

Animal studies were conducted according to protocols approved by the San Raffaele Scientific Institute (Milan, Italy) Institutional Animal Care and Use Committee (IACUC) and adhered strictly to the Italian Ministry of Health guidelines for the use and care of experimental animals.

Research on the bacterial isolates from the individual with CF has been approved by the responsible physician at the CF center at Hannover Medical School, Germany. All patients gave informed consent before the sample collection. Approval for storing of biological materials was obtained by the Hannover Medical School, Germany.

### Bacterial strains

The *P. aeruginosa* PAO1/PAO1293 STM library was described previously [22]. PAO1293 is a derivative of PAO1 from the Holloway laboratory [45] and carries the E79tv-2 giving chloramphenicol susceptibility. STM mutants were cultured at 37°C in brain-heart infusion (BHI) or on TSB agar plates containing 300 µg/ml kanamycin or 25 µg/ml tetracycline. For agar beads preparation, STM mutants were grown in BHI broth in a 96-well plate at 37°C for 18h, pooled, centrifuged at 4000 rpm for 10 min and re-suspended at a concentration of approximately 5×10^9^ cfu/ml. *P. aeruginosa* clinical strains were isolated from CF patients as described previously [5] [1].

### Pos-STM screening in a mouse model of chronic infection

C57Bl/6NCrlBR (Charles River) (6–8 weeks) male mice were infected with STM mutant loads ranging from 5×10^5^ and 2.5×10^6^ CFUs of *P. aeruginosa* embedded in agar beads and infected as previously described [23]. Fourteen days after infection, murine lungs were excised, homogenized and plated onto TSB-agar plates for cfu counting and identification. For STM mutant identification, at least >10^4^ bacterial colonies grown on agar plates were pooled from each positive output pools and subjected to chromosomal DNA extraction and multiplex PCR analysis according to established protocol [22]. For confirmation, the PCR was repeated for each putative STM mutant.

### Histological examination and immunofluorescence

Lungs were removed, fixed in 10% buffered formalin for at least 24 h and embedded in paraffin. Consecutive 2-µm sections from the middle of the five lung lobes were used for histological and immunofluorescence examination in each mouse. Sections for histological analysis were stained by Haematoxylin-Eosin and examined blindly. Localization of *P. aeruginosa* was performed in de-paraffinized lung sections by employing a rabbit antiserum specific for *P.aeruginosa* and Texas Red-labelled goat anti-rabbit IgG as described [1]. Immunofluorescence images were recorded with an EM-CCD Hamamatsu C9100 CCD camera (Hamamatsu Photonics, Hamamatsu City, Japan) mounted on an UltraVIEW Spinning Disk Confocal Microscope (Perkin Elmer, Waltham, MA, USA). Slides stained with haematoxylin and eosin were visualized with Axioplan2 (Zeiss, Jena, Germany) with AxioCam provided with the CCD MRc5 (Zeiss).

### Sequencing transposon flanking regions

Transposon flanking regions were amplified by the Y-linker method [46]. Approximately 40 ng of genomic DNA, digested with *Nla*III, was ligated to 1 µg of Y linker with T4 DNA ligase in a final volume of 20 µl. After overnight incubation at 16°C, 1 µl of the reaction mixture was used as template for hot-start PCR amplification, using a transposon specific oligos (pUTKana2; pUTgfpR2) [22] and the Y-linker specific Y-primer oligo (**Table S1** in **Text S1**). The PCR product was purified from agarose gel with QIAquick Gel Extraction Kit (Qiagen) and then sequenced by Eurofins MWG-Operon (Germany).

### Phenotypic characterization

Motility and analysis for LasR mutants were evaluated as described [28] [7]. Mutation frequency measurement were determined by rifampicin assay [10] [47]. Protease and siderophore secretion, pyocyanin, mucoidy, and biofilm formation assay haemolytic/autolytic activity were assayed as described in the online data supplement.

### Gene sequencing and computational tools

*P. aeruginosa* clinical strains described previously [5] [1] were used to sequence the genes inserted in the Pos-STM mutants. PCR genes amplification was carried out using the primers listed in **Table S1** in **Text S1**. The amplified DNA samples were sequenced by standard automated DNA sequence technology. The sequence results were compared within *P. aeruginosa* clonal lineages with BioEdit v7.0.5 to determine the occurrence of sequence variants within the selected genes. The program SIFT was used to predict the effect of non-synonymous mutations on protein function [44].

### Cell cultures and invasion assay

The A549 (human type II pneumocytes) cell line was purchased from ATCC CCL-185 and cultured as described [48]. Bacteria invasion assay was performed using ceftazidime-amikacin protection assay with minor modifications [48]. *P. aeruginosa* strains, grown to the mid-exponential phase, were used to infect cell monolayers at a 100∶1 multiplicity of infection for 2h. The monolayers were washed with PBS, treated with antibiotic for 2 h, washed, lysed with H_2_O and plated on TSB-agar plates (Difco).

### IL-8 secretion

IL-8 was determined in supernatants collected from the cell cultures described above using an ELISA kit (Biosource Europe). According to the manufacturer the sensitivity of the assay is less than 0.7 pg/ml. Values were normalized to 10^6^ cells; results were expressed as mean ± SD.

### Statistical analysis

Statistical calculations and tests were performed using Student's t-test and the Chi-square test considering p<0.05 as the limit of statistical significance. All data were expressed as mean +/− standard deviation (SD).

## Supporting Information

Text S1 Material and methods concerning phenotypic and genetic characterization of Pos-STM mutants, list of primers used for identification and sequencing of *P. aeruginosa* Pos-STM mutants and clinical strains (Table S1), virulence of *P. aeruginosa* Pos-STM mutants in a murine model of airways infection (Table S2), Classification of Pos-STM inserted genes by molecular function (Table S3), Phenotypic characterization of *P. aeruginosa* Pos-STM mutants and interaction with human cells (Table S4), Pos-STM genes in which mutations occurred during chronic airways infection in CF patients (Table S5).
(0.29 MB DOC)
