# Supplementary material for: Positive Signature-Tagged Mutagenesis in Pseudomonas aeruginosa: Tracking Patho-Adaptive Mutations Promoting Airways Chronic Infection
Source: PLoS Pathog. 2011 Feb 3;7(2):e1001270. doi: 10.1371/journal.ppat.1001270 (PMC3033382; doi:10.1371/journal.ppat.1001270)
Supplement: Text S1 — Material and methods concerning phenotypic and genetic characterization of Pos-STM mutants, list of primers used for identification and sequencing of P. aeruginosa Pos-STM mutants and clinical strains (Table S1), virulence of P. aeruginosa Pos-STM mutants in a murine model of airways infection (Table S2), Classification of Pos-STM inserted genes by molecular function (Table S3), Phenotypic characterization of P. aeruginosa Pos-STM mutants and interaction with human cells (Table S4), Pos-STM genes in which mutations occurred during chronic airways infection in CF patients (Table S5). (0.29 MB DOC) [file ppat.1001270.s001.doc]

**Positive signature-tagged mutagenesis in *Pseudomonas aeruginosa*: tracking patho-adaptive mutations promoting airways chronic infection**

Irene Bianconi, Andrea Milani, Cristina Cigana, Moira Paroni, Roger C. Levesque Giovanni Bertoni, Alessandra Bragonzi

**Supporting Information**

**MATERIALS AND METHODS**

***Phenotypic characterization of Pos-STM mutants*.**

***Motility assay.*** Swarm agar was based on TSB solidified with 0.5% agar, twich agar was instead solidified with 1% agar. Single colonies of bacteria were picked and inoculated on agar plates and incubated for 15 h at 37°C for swarming test and 16 h at 37°C plus an additional 48 h at RT for twitching test. Motility was indicated by the formation of an halo around the colony [1].

***Protease secretion***. 2 μl of overnight cultures were inoculated onto skim milk plates solidified with 1 % agar and incubated overnight at 37°C. Protease secretion was indicated by a transparent halo around the colony.

***Siderophore secretion.*** 200 ml of CAS stock solution were prepared and mixed with 10 ml of Fe(III)Cl3 solution. 145.8 mg of HDTMA dissolved in 80 ml of water were added to give a dark blue solution which was autoclaved at 121°C for 30 min. Piperazin diethansulfonic acid (PIPES) agar was prepared from (10x) MM9 solution. The solution was filled up to 870 ml with distilled water and supplemented with of 1 % agar. 30 ml (10x) LB medium, 2 ml glycerol, 2 ml 1 M Na2SO4, 1 ml 0.1 M CaCl2 and 100 ml CAS stock solution were added to the PIPES agar under sterile conditions and poured into Petri dishes. 2 μl of overnight cultures were inoculated onto the plates and incubated at 37°C for 24 h. An orange halo indicated siderophore production.

***Pyocyanin assay.*** Bacteria were grown in liquid King Medium A (20 g/l bactopeptone, 15 mM MgCl_2_, 56 mM K_2_SO_4_, 1% glycerol) with aeration. After 26 h of incubation, OD_600_ of each culture was measured and then cells were collected by centrifugation at 5000 rpm for 5 min. Pyocyanin production levels was determined measuring OD_695_ of the supernatant and normalizing to OD_600_.

***Biofilm formation assay.*** Biofilm formation was assessed by the crystal violet staining assay. Briefly, *P. aeruginosa* was grown in LB medium and normalized to an OD_600_ of 0.2 in 200 μl of the same medium in a 96-well U-bottom microtiter plate (Greiner Bio-One). After 18 h of static incubation at 37 °C, the liquid culture was removed and OD_600_ was measured to determine the cell density of planktonic bacteria. Cells attached to the microtiter wells through biofilm were washed twice with water and then stained with 1% crystal violet. After 30 min of staining, the cells were washed again with water, dried and then re-suspended in 0.2 ml of ethanol 95% by pipette scraping. The OD_600_ of re-suspended biofilm cells was determined and normalized to the OD_600_ of corresponding planktonic cells.

***Haemolytic/autolytic activity.*** 2 μl of overnight cultures were inoculated onto blood-agar plates (BD Biosciences) and incubated for 48 h at 37°C. Haemolysis was indicated by an orange halo around the colony, autolysis can be seen in the middle of the colony

***Mutation frequency measurement***. A single *P. aeruginosa* colony was grown in 5 ml of TSB overnight at 37°C. Thereafter, aliquots from serial dilutions were plated on TSB-agar plates with and without rifampicin (300 μg/ml) and counted after 36 h at 37°C. The mutation frequencies on rifampicin were determined relative to the total count of viable organisms plated according to previously established criteria [2, 3].

***Analysis of LasR phenotypes***. Colony surface iridescent and the metallic sheen indicates a phenotype that specifically identifies the LasR mutation. It was analysed as described previously [4].

***Southern blotting analysis of Pos-STM mutants.*** Chromosomal DNA was prepared from overnight cultures of Pos-STM mutants grown in BHI at 37°C and digested with a prolonged incubation with either *PstI* or *XhoI* restriction enzymes. Digested chromosomal DNAs were then analyzed by Southern blotting using [α-^32^P]-CTP-labelled ribo-probes transcribed using as a template internal portions of Km^R^ and Tc^R^ genes of mini-Tn5Km2 and miniTn5Tc, respectively.

**REFERENCES**

1. O'Toole GA, Kolter R. (1998) Flagellar and twitching motility are necessary for Pseudomonas aeruginosa biofilm development. Mol Microbiol 30: 295-304.

2. Oliver A, Cantón R, Campo P, Baquero F, Blázquez J (2000) High frequency of hypermutable Pseudomonas aeruginosa in cystic fibrosis lung infection. Science 288: 1251-1254.

3. Montanari S, Oliver A, Salerno P, Mena A, Bertoni G, et al. (2007) Biological cost of hypermutation in Pseudomonas aeruginosa strains from patients with cystic fibrosis. Microbiology 153: 1445-1454.

4. D'Argenio DA, Wu M, Hoffman LR, Kulasekara HD, Déziel E, et al (2007) Growth phenotypes of Pseudomonas aeruginosa lasR mutants adapted to the airways of cystic fibrosis patients. Mol Microbiol 64: 512-533.

5. Bragonzi A, Wiehlmann L, Klockgether J, Cramer N, Worlitzsch D, et al. (2006) Sequence diversity of the mucABD locus in Pseudomonas aeruginosa isolates from patients with cystic fibrosis. Microbiology 152: 3261-3269.

6. Bragonzi A, Paroni M, Nonis A, Cramer N, Montanari S, et al. (2009) Pseudomonas aeruginosa microevolution during cystic fibrosis lung infection establishes clones with adapted virulence AJRCCM 180: 138-145.

**Table S1: List of primers used for identification and sequencing of *P. aeruginosa* Pos-STM mutants and clinical strains.**

| **ORF** | **Primer name** | **Sequenza 5’->3’** |
| --- | --- | --- |
|  | Y-linker_1 | TTTCTGCTCGAATTCAAGCTTCTAACGATGTACGGGGACACATG |
|  | Y-linker_2 | TGTCCCCGTACATCGTTAGAACTACTCGTACCATCCACAT |
|  | Y-primer | CTGCTCGAATTCAAGCTTCT |
| PA0410 | PA0410_for | AGAGACCCTGCTGAAAACCAT |
|  | PA0410_rev | AACCGGTGAAGCTGTTTATACG |
| PA0436 | PA0436_for | ATTTGCTGGTAGCCTTGGT |
|  | PA0436_rev | CAGACCACGGTGTTCCTC |
| PA0437 | PA0437_for | GCCTACCTGTTGCTCAATCC |
|  | PA0437_rev | TACCAGGTCGAGGTGCTCA |
| PA0499 | PA0499_for | CACATTCAGGAATATGAATGGAAA |
|  | PA0499_rev | GGTAATTGCATTCATGGTGTATTC |
| PA0890 | PA0890_for | CGAGCCGTTCACCTACTACCTC |
|  | PA0890_rev | CTGGACTGGATGTAGACCTTGC |
| PA0895 | PA0895_for1 | CTACAATCCTTTCATCGATAGC |
|  | PA0895_rev1 | GTAGTGCATGTAGGCGAACAG |
|  | PA0895_for2 | GCGATCTCCGACAAGACCTG |
|  | PA0895_rev2 | GAACGTCCAGCAGGGAAGAT |
| PA1077 | PA1077_for | CGGGTTACCCTTAAATAAACTCAA |
|  | PA1077_rev | CTACCGGCAATGTTGAAGACACT |
| PA1856 | PA1856_for1 | TACCCAGAAAAATACCGATAAC |
|  | PA1856_rev1 | AGGAGGATCAGCGACATCAC |
|  | PA1856_for2 | CCTCGGCATGATGTACTACT |
|  | PA1856_rev2 | GGCAGATCTTGCCGGTATCC |
| PA2252 | PA2252_for1 | ATTGTAACCAGGTGCGTCAT |
|  | PA2252_rev1 | ACGAAGGTGTCGAGGAACAC |
|  | PA2252_for2 | GCGTCACCCTGTACGTCATC |
|  | PA2252_rev2 | GTGTAGAGCACGAAGAGGTTCT |
| PA2972 | PA2972_for1 | GTCAAAGGAAATTAGCCATAAACC |
|  | PA2972_rev1 | CTGAGGGTGTTTCCCTTCCTTAT |
| PA2998 | PA2998_for | GATTCTGCGGGACAATCTG |
|  | PA2998_rev | CAGGGCTACCAGCAGTGTG |
| PA3478 | PA3478_for1 | TTGCATAACGCACGGAGTAG |
|  | PA3478_rev1 | AAAAAGCCTCCGTCATTCCT |
|  | PA3478_for2 | AACCCTTGACCTGCGAAGAC |
|  | PA3478_rev2 | GCATTGTCGAACTGGTCGTG |
| PA4554 | PA4554_for | GCATCGGCACCTTCTACTAC |
|  | PA4554_rev | TTCTTCTCGACCTGGACTTC |
| PA4842 | PA4842_for1 | TTATGCCTGTTTCGGAAAAT |
|  | PA4842_rev1 | GAGTTGAACAGCAGGATGAA |
|  | PA4842_for2 | CAGATGGTCACCGGCTACAC |
|  | PA4842_rev2 | GCGCGGGAAATGTAGTTG |
| PA4887 | PA4887_for1 | CTTCCCGAATCTAACAATGCTC |
|  | PA4887_rev1 | GCCTGTTCGACCGTTTCTAC |
|  | PA4887_for2 | TGAACCAGTACATGGTGATCG |
|  | PA4887_rev2 | CATCGCCACGTGTTGCAG |
| PA5028 | PA5028_for | TCTATGTCAGGACCGAGACG |
|  | PA5028_rev | GTCCTGGTTACCTCGTAGAAGC |
| PA5053 | PA5053_for | CCCGCAGTGTGGAGATTT |
|  | PA5053_rev | GCGTCATGGACATTCGTTTA |

**Table S2. Virulence of *P. aeruginosa* Pos-STM mutants in a murine model of airways infection*.***

| **STM mutant** | **Mortality** ^#^ | **Chronic infection^§^** | **Cfu/lung^¶^** | **P value*** |
| --- | --- | --- | --- | --- |
|  |  |  |  | **(Chronic infection)** |
|  |  |  |  |  |
| PAO1293 *wt* | 20% (2/10) | 12.5% (1/8) | 8.9x10^3^ |  |
| 4T24K | 33.3% (5/15) | 70% (7/10)* | 2.6x10^3^ | 0.0248 |
| 70T22T | 33.3% (3/9) | 100% (6/6)* | 1.9x10^4^ | 0.0047 |
| 4T9G | 33% (3/9) | 100% (6/6)* | 4.2x10^4^ | 0.0047 |
| 13T9G | 50% (5/10) | 80% (4/5)* | 3.1x10^4^ | 0.0319 |
| 70T15K | 27.3% (3/11) | 100% (8/8)* | 1.8x10^4^ | 0.0014 |
| 13T22K | 22.2% (2/9) | 71.4% (5/7)* | 8.3x10^3^ | 0.0406 |
| 22T18G | 20.0% (2/10) | 75.0% (6/8)* | 4.2 x10^3^ | 0.0406 |
| 31T23K | 11.1% (1/9) | 100% (8/8)* | 5.5x10^3^ | 0.0014 |
| 13T18G | 38.5% (5/13) | 75 % (6/8)* | 2.3x10^3^ | 0.0406 |
| 4T16K | 46.1% (6/13) | 71.4% (5/7)* | 1.8x10^3^ | 0.0406 |
| 4T5T | 20% (2/10) | 75 % (6/8)* | 2.9x10^4^ | 0.0406 |
| 22T13K | 22.2% (2/9) | 71.4% (5/7)* | 2.4x10^4^ | 0.0406 |
| 70T5K | 53.3% (8/15) | 85.7% (6/7)* | 2.3x10^3^ | 0.0101 |
| 31T4T | 30.7% (4/13) | 77.8% (7/9)* | 1.4x10^3^ | 0.0152 |
| 22T4G | 10% (1/10) | 88.8% (8/9)* | 1.3x10^4^ | 0.0034 |
| 22T9K | 33% (3/9) | 83.3% (5/6)* | 1.9x10^4^ | 0.0256 |

^#^ Mortality was calculated as no. of dead/total mice during the first three days of infection.

^§^ Chronic infection was calculated as no of infected mice/total mice in surviving 14 days after challenge.

**^¶^** Bacterial load in the lung of surviving mice 14 days after challenge.

^*^ Statistical significance by Chi square analysis (P<0.05) of the STM mutants versus the wild-type PAO1293.

**Table S3: Classification of Pos-STM inserted genes by molecular function.**

| **STM name** | **Inserted *locus***^§^ | **Gene name** | **Class** | **Gene annotation** | **Possible polar effects of the insertion** | **Similarity** |
| --- | --- | --- | --- | --- | --- | --- |
| 4T24K | PA0410 | *pilI* | 1 | Twitching motility protein PilI; | - |  |
|  |  |  | Motility & Attachment | last gene of operon ID:85* |  |  |
| 4T9G | PA0499 | - | 3 | Probable pilus assembly protein, chaperone; | + | 47% filamentous hemagglutinin *Bordetella pertussis* |
|  |  |  | Chaperones & heat shock proteins | first gene of operon ID:108 |  |  |
| 13T9G | PA0890 | *aotM* | 1 | Arginine/ornithine transport protein AotM; | + |  |
|  |  |  | Transport of small molecules | second gene of operon ID:193 |  |  |
| 70T15K | PA0895 | *aruC* | 1 | N-succinylglutamate 5-semialdehyde dehydrogenase; single gene | - |  |
|  |  |  | Amino acid biosynthesis & metabolism |  |  |  |
| 13T22K | PA1077 | *flgB* | 2 | Flagellar basal-body rod protein FlgB; | + | 62% FlgB *Salmonella typhimurium* |
|  |  |  | Motility & Attachment | first gene of operon ID:233 |  |  |
| 22T18G | PA1856 | - | 3 | Probable cytochrome oxidase subunit; | + | 74% CytN *Azospirillum brasilense* |
|  |  |  | Putative enzymes | first gene of operon ID:390 |  |  |
| 31T23K | PA2252 | - | 3 | Probable AGCS sodium/alanine/glycine symporter; | - | 64% AlsT *Bacillus subtilis* |
|  |  |  | Transport of small molecules | single gene |  |  |
| 13T18G | PA2972 | - | 4 | Conserved hypothetical protein; | - | 70% YceF Escherichia coli |
|  |  |  | Hypothetical, unclassified, unknown | single gene |  |  |
| 4T16K | PA2998 | *nqrB* | 2 | Na+-translocating NADH:ubiquinone oxidoreductase subunit Nrq2; | + | 76% NqrB *Vibrio alginolyticus* |
|  |  |  | Energy metabolism | second gene of the operon ID:617 |  |  |
| 4T5T | PA3478 | *rhlB* | 1 | Rhamnosyltransferase chain B; | - |  |
|  |  |  | Secreted Factors (toxins, enzymes, alginate) | single gene |  |  |
| 22T13K | PA4554 | *pilY1* | 1 | Type 4 fimbrial biogenesis protein PilY1; | + |  |
|  |  |  | Motility & Attachment | fifth gene of the operon ID:932 |  |  |
| 70T5K | PA4842 | - | 4 | Hypothetical protein; | + |  |
|  |  |  | Hypothetical, unclassified, unknown | first gene of the operon ID:994 |  |  |
| 31T4T | PA4887 | - | 3 | Probable major facilitator superfamily (MFS) transporter; | - | 47% YcaD *E. coli* |
|  |  |  | Transport of small molecules | single gene |  |  |
| 22T4G | PA5028 | - | 4 | Conserved hypothetical protein; | - | 51% SoJ *Bacillus subtilis* |
|  |  |  | Hypothetical, unclassified, unknown | last gene of the operon ID:1038 |  |  |
| 22T9K | PA5053 | *hslV* | 2 | Heat shock protein HslV; | + | 100% hslV *E. coli* |
|  |  |  | Chaperones & heat shock proteins | first gene of the operon ID:1044 |  |  |
| 70T22T | PA0436-PA0437 | - | Intergenic region | Intergenic region located between convergent ORFs | - |  |

* ID operon according with DOOR (Database of prokaryotic operons)

^§^ Indicates the gene name assigned by the annotation and sequencing group at the http://www.pseudomonas.com.

**Table S4: Phenotypic characterization of *P. aeruginosa*** Pos-STM mutants and interaction with human cells.

| **PA number** | **Gene name** | **Swimming**  zone ø cm | **Twitching**  zone ø cm | **Pyocyanin**  (OD_695_ 26h) | **Biofilm** | **Invasion of A549 cells^§^** | **Secretion of IL-8^§^** |
| --- | --- | --- | --- | --- | --- | --- | --- |
|  |  |  |  |  |  |  |  |
| PAO1293 |  | 1.5 | 2.6 | 0.09±0.03 | 1.37±0.60 | 1 | 1 |
| PA0410 | *pilI* | - | - | 0.10±0.06 | 1.70±0.79 | 3.43±1.22* | 1.24±0.55 |
| PA0499 | - | 1.4 | 2.6 | 0.08±0.02 | 1.48±0.43 | 10.40±2.30* | 1.04±0.17 |
| PA0890 | *aotM* | 1.8 | 1.8 | 0.07±0.04 | 2.28±0.48* | 2.82±1.93 | 0.85±0.12 |
| PA0895 | *aruC* | 1.5 | 1.3 | 0.12±0.07 | 1.54±0.58 | 9.44±2.97* | 1.00±0.25 |
| PA1077 | *flgB* | - | 1.0 | 0.16±0.15 | 0.76±0.18* | 9.55±4.97* | 0.92±0.11 |
| PA1856 | - | 1.7 | 1.9 | 0.04±0.02 | 2.01±0.67 | 4.60±1.16* | 0.81±0.21 |
| PA2252 | - | 1.2 | 1.7 | 0.05±0.04 | 1.32±0.24 | 7.24±3.85* | 0.83±0.04* |
| PA2972 | - | 1.7 | - | 0.04±0.05 | 1.55±0.28 | 1.32±0.83 | 0.89±0.15 |
| PA2998 | *nqrB* | 1.5 | 2.0 | 0.10±0.02 | 1.37±0.45 | 9.24±1.50* | 0.83±0.05* |
| PA3478 | *rhlB* | 2.3 | 1.5 | 0.10±0.04 | 2.39±0.54* | 1.25±1.17 | 0.78±0.10* |
| PA4554 | *pilY1* | - | - | 0.03±0.02* | 0.72±0.37* | 0.28±0.34* | 1.48±0.42 |
| PA4842 | - | 1.5 | 1.5 | 0.04±0.02* | 1.92±0.41 | 15.42±3.61* | 0.93±0.01* |
| PA4887 | - | 1.7 | 1.1 | 0.10±0.02 | 1.81±0.38 | 8.10±3.30* | 1.08±0.28 |
| PA5028 | - | 1.3 | 2.3 | 0.10±0.10 | 1.90±0.63 | 2.80±1.78 | 0.71±0.17* |
| PA5053 | *hslV* | 1.3 | 2.4 | 0.10±0.09 | 1.95±0.35 | 12.21±5.38* | 1.00±0.11 |
| PA0436 -PA0437 | - | 1.8 | 1.3 | 0.09±0.07 | 1.22±0.28 | 5.55±1.33* | 0.77±0.25 |

*Significant differences of Pos-STM mutants with PAO1293 are reported. Statistical calculations were performed using Student’s t-test.

None of the Pos-STM mutants differed with regard to mucoidy, hypermutability, protease, siderophore, autolysis, hemolytic activity, and LasR colony morphology when compared with PAO1293.

^§^ Fold of invasion of Pos-STM mutants in A549 cells and secretion of IL-8 relative to PAO1293.

**Table S5: Pos-STM genes in which mutations occurred during chronic airways infection in CF patients.**

| **STM name** | **PAO1 annotation** | **Gene name** | **Gene function** | **Functional category** | **Strains^§^** | **Type of mutation*** | **Nucleotidic mutation** | **Aminoacidic mutation** |
| --- | --- | --- | --- | --- | --- | --- | --- | --- |
| 13T9G | PA0890 | aotM | arginine/ornithine transport protein AotM | Membrane proteins.  Transport of small molecules | KK1 vs KK72 | synonymous | A120 - G |  |
|  |  |  |  |  |  | synonymous | T174 - C |  |
| 70T15K | PA0895 | aruC | N-succinylglutamate 5-semialdehyde dehydrogenase | Amino acid biosynthesis and metabolism | TR1 vs TR67 | non synonymous | G590 - T | G197 – V^#^ |
| 22T18G | PA1856 | - | probable cytochrome oxidase subunit | Putative enzymes | AA2 vs AA43-AA44 | synonymous | G105 - C |  |
|  |  |  |  |  |  | synonymous | G126 - A |  |
|  |  |  |  |  |  | synonymous | C288 - T |  |
|  |  |  |  |  |  | non synonymous | T290 - C | V97 - A |
|  |  |  |  |  |  | synonymous | C363 - T |  |
|  |  |  |  |  |  | synonymous | G378 - A |  |
|  |  |  |  |  |  | synonymous | A399 - G |  |
|  |  |  |  |  |  | synonymous | C417 - G |  |
|  |  |  |  |  |  | synonymous | C465 - T |  |
|  |  |  |  |  |  | non synonymous | C540 - G | I180 - M |
|  |  |  |  |  | BT2 vs BT72-BT73 | synonymous | A121 – G |  |
|  |  |  |  |  |  | synonymous | T288 – C |  |
|  |  |  |  |  |  | non synonymous | C290 – T | A97 - V |
|  |  |  |  |  |  | synonymous | T363 – C |  |
|  |  |  |  |  |  | synonymous | A378 – G |  |
|  |  |  |  |  |  | synonymous | G399 – A |  |
|  |  |  |  |  |  | synonymous | T465 – C |  |
|  |  |  |  |  |  | non synonymous | G540 – C | M180 - I |
|  |  |  |  |  | TR1 vs TR66-TR67 | synonymous | C67 – T |  |
|  |  |  |  |  |  | synonymous | C105 – G |  |
| 31T23K | PA2252 | - | probable AGCS sodium/alanine/glycine symporter | Transport of small molecules | AA2 vs AA43 | non synonymous | T11 - A | M4 - K |
|  |  |  |  |  | KK1 vs KK72 | synonymous | T61 – C |  |
|  |  |  |  |  |  | synonymous | T90 – C |  |
|  |  |  |  |  |  | synonymous | A129 – G |  |
|  |  |  |  |  |  | synonymous | G141 – A |  |
|  |  |  |  |  |  | synonymous | C579 – T |  |
|  |  |  |  |  |  | synonymous | C642 – T |  |
|  |  |  |  |  |  | synonymous | A713 – C |  |
|  |  |  |  |  |  | synonymous | G744 – A |  |
|  |  |  |  |  |  | synonymous | A853 – G |  |
|  |  |  |  |  |  | synonymous | A1098 – G |  |
|  |  |  |  |  |  | synonymous | C1254 – T |  |
|  |  |  |  |  |  | synonymous | T1293 – C |  |
|  |  |  |  |  |  | synonymous | A1323 – G |  |
|  |  |  |  |  |  | non synonymous | G1391 – A | R464 - H |
|  |  |  |  |  |  | non synonymous | A1339 – C | N480 - T |
| 13T18G | PA2972 | - | conserved hypothetical protein | Hypothetical, unclassified, unknown | MF1 vs MF51 | synonymous | C27 - T |  |
| 4T5T | PA3478 | rhlB | rhamnosyltransferase chain B | Secreted factors (toxins, enzymes, alginate) | KK1 vs KK71-KK72 | non synonymous | C603 - G | H201^#^ - Q |
| 31T4T | PA4887 | - | probable major facilitator superfamily (MFS) transporter | Membrane proteins. Transport of small molecules | SG1 vs SG57 | non synonymous | A52 – G  A53 – T | K18^#^ - V^#^ |
|  |  |  |  |  | SG1 vs SG58 | non synonymous | A53 – T | K18^#^ - M |
|  |  |  |  |  |  | non synonymous | C634 – A | L212 - M |

^§^ Strains were collected at the onset of chronic colonization (numbered 1-2 per patient) or after 4-18 years of chronic airway colonization (numbered 43-73) [5, 6].

* Alignment of early versus late *P. aeruginosa* isolates from CF single patients.

^#^ Non-synonymous mutations that are predicted to be non-tolerated by computational prediction .
